# Differential Expression of Circadian Clock Genes in the Bovine Neuroendocrine Adrenal System

**DOI:** 10.3390/genes14112082

**Published:** 2023-11-15

**Authors:** Audrey L. Earnhardt-San, Emilie C. Baker, David G. Riley, Noushin Ghaffari, Charles R. Long, Rodolfo C. Cardoso, Ronald D. Randel, Thomas H. Welsh

**Affiliations:** 1 Department of Animal Science, Texas A&M University, College Station, TX 77843, USA; audrey.san@delval.edu (A.L.E.-S.); ebaker@wtamu.edu (E.C.B.); david.riley@ag.tamu.edu (D.G.R.); r.cardoso@tamu.edu (R.C.C.); 2 Texas A&M AgriLife Research Center, Overton, TX 75684, USA; charles.long@ag.tamu.edu (C.R.L.); ron.randel@ag.tamu.edu (R.D.R.); 3 Department of Computer Science, Prairie View A&M University, Prairie View, TX 77070, USA; noghaffari@pvamu.edu

**Keywords:** circadian, clock genes, peripheral clock, bovine, stress axis tissues

## Abstract

Knowledge of circadian rhythm clock gene expression outside the suprachiasmatic nucleus is increasing. The purpose of this study was to determine whether expression of circadian clock genes differed within or among the bovine stress axis tissues (e.g., amygdala, hypothalamus, pituitary, adrenal cortex, and adrenal medulla). Tissues were obtained at an abattoir from eight mature nonpregnant Brahman cows that had been maintained in the same pasture and nutritional conditions. Sample tissues were stored in RNase-free sterile cryovials at −80 °C until the total RNA was extracted, quantified, assessed, and sequenced (NovaSeq 6000 system; paired-end 150 bp cycles). The trimmed reads were then mapped to a *Bos taurus* (*B. taurus*) reference genome (Umd3.1). Further analysis used the edgeR package. Raw gene count tables were read into RStudio, and low-expression genes were filtered out using the criteria of three minimum reads per gene in at least five samples. Normalization factors were then calculated using the trimmed mean of M values method to produce normalized gene counts within each sample tissue. The normalized gene counts important for a circadian rhythm were analyzed within and between each tissue of the stress axis using the GLM and CORR procedures of the Statistical Analysis System (SAS). The relative expression profiles of circadian clock genes differed (*p* < 0.01) within each tissue, with neuronal PAS domain protein 2 (*NPAS2*) having greater expression in the amygdala (*p* < 0.01) and period circadian regulator (*PER1*) having greater expression in all other tissues (*p* < 0.01). The expression among tissues also differed (*p* < 0.01) for individual circadian clock genes, with circadian locomotor output cycles protein kaput (*CLOCK*) expression being greater within the adrenal tissues and nuclear receptor subfamily 1 group D member 1 (*NR1D1*) expression being greater within the other tissues (*p* < 0.01). Overall, the results indicate that within each tissue, the various circadian clock genes were differentially expressed, in addition to being differentially expressed among the stress tissues of mature Brahman cows. Future use of these findings may assist in improving livestock husbandry and welfare by understanding interactions of the environment, stress responsiveness, and peripheral circadian rhythms.

## 1. Introduction

During a 24-h period, several physiological and behavioral processes are controlled by a natural internal process known as a circadian rhythm. These endogenous rhythms are adaptable as they can be synchronized with external environmental conditions to allow the organism to have optimal performance [1]. The cooperative expression of many genes is essential for these physiological processes, whose regulation is dependent on the environmental and genetic control of circadian rhythms. These rhythms also play an important role in regulating stress, shown through the regulation of glucocorticoid secretion from the adrenal gland in a 24 h cycle [2,3,4].

The control of circadian rhythms in mammals is primarily located within the brain in the suprachiasmatic nuclei (SCN), which is a cluster of neurons in the anterior part of the hypothalamus [5,6]. This central pacemaker serves to synchronize the intrinsic circadian clocks found throughout other regions of the brain as well as peripheral tissues and organs and is dependent upon the transcription of several core clock genes listed in Table 1 [6,7]. The first major clock genes involved are circadian locomotor output cycles protein kaput (*CLOCK*) and aryl hydrocarbon receptor nuclear translocator like (*ARNTL*), which form a heterodimer together that allows them to directly bind to DNA regulatory elements and induce the transcription of other core clock genes. The clock gene targets of this heterodimer include period circadian regulator (*PER1* and *PER2*) and cryptochrome circadian regulator (*CRY1* and *CRY2*), which encode for proteins that can also heterodimerize and initiate negative feedback on their own transcription by translocating into the nucleus and directly blocking the CLOCK-ARNTL heterodimer [6,8]. Additionally, the CLOCK-ARNTL heterodimer induces the rhythmic transcription of nuclear receptor subfamily 1 group D member 1 (*NR1D1*), which produces the nuclear receptor REV-ERBα that is also responsible for creating negative feedback by repressing *CLOCK* and *ARNTL* gene expression [8,9]. These various feedback loops generate a rhythmic cycle of transcription that involves phases of expression dependent on cis-regulatory elements of the target genes [9]. If production of CLOCK is deficient or absent, then a paralog of this core clock gene, neuronal PAS domain protein 2 (*NPAS2*), can be a functional substitute within the SCN and peripheral tissues to continue regulating circadian rhythms [9,10].

Circadian and stress-mediated systems play an important role in regulating the tissues of the stress axis, as it has been shown that both the hypothalamic-pituitary-adrenal (HPA) axis and sympathomedullary system receive strong circadian input. The endocrine physiology regulated by the paraventricular nucleus (PVN) of the hypothalamus and anterior pituitary (PIT) exhibits rhythmic properties controlled by circadian rhythms and their associated genes [11,12]. Processing of fearful and threatening stimuli and memory formation within the amygdala (AMYG) are dependent on circulating glucocorticoids for circadian regulation [13,14,15]. Fluctuations in circulating glucocorticoids along with the regulation of stress are influenced by circadian rhythms.

Despite the importance of circadian rhythm mediation of reproduction [16,17], lactation [18,19], stress response [20,21], immune function [22], and metabolism [23,24], the expression of circadian clock genes had yet to be investigated within the stress axis tissues of cattle at the inception of this study. The purpose of this study was to (1) demonstrate circadian clock gene expression in the stress axis tissues of cattle and (2) determine whether expression of circadian clock genes differed within or among neuroendocrine tissue components of the bovine stress axis (e.g., the amygdala, hypothalamus, and pituitary and adrenal glands).

## 2. Materials and Methods

### 2.1. Animal Procedures

All procedures followed the Guide for the Care and Use of Agricultural Animals in Research and Teaching [25] and were approved by the Texas A&M AgriLife Research Animal Use and Care Committee. Mature nonpregnant Brahman cows (*n* = 8; 5 years of age) were maintained in the same pasture and nutritional conditions at the Texas A&M AgriLife Research & Extension Center at Overton. The cows were transported to the Texas A&M University Department of Animal Science Beef Cattle Center and held for approximately 15 h in pens with free access to water before being transported to the departmental abattoir (the Rosenthal Meat Science & Technology Center) and harvested humanely between 800 and 1100 h. The sample tissues were promptly isolated. The sample tissues, including the paraventricular nucleus (PVN), anterior pituitary (PIT), amygdala (AMYG), adrenal cortex (ADC), and adrenal medulla (ADM), were processed on site, stored in RNase-free sterile cryovials, snap-frozen in a portable liquid nitrogen tank, and then moved to the research laboratory in an adjacent building for storage at −80 °C.

### 2.2. Sample Preparation and RNA Extraction

Tissue containing the PVN was collected from frozen blocks of tissue containing the septum, preoptic area, and hypothalamus of the brain, which were cut into coronal sections of 20 μm using a cryostat and thaw-mounted on glass microscope slides. A single series of tissue sections from the hypothalamus containing sections 200 μm apart was processed for Cresyl violet staining and observed using bright-field microscopy to determine the location of the paraventricular nucleus. The location of the PVN was determined based upon identification of well-established anatomical landmarks for rat [26] and sheep [27] brains, including the third ventricle and the anterior commissure. A separate series of sections containing the PVN was used for tissue dissection and RNA isolation. An area approximately 1 mm in diameter encompassing the PVN was scraped from the slides using a sterile 25-gauge needle. The sections were kept frozen during scraping and based on a pilot study to evaluate the RNA integrity and concentration, 12 sections from each animal were used for extraction isolation of at least 1–4 μg total RNA for sequencing procedures. The scraped tissue was placed immediately into 500 μL of chilled TRIzol Reagent (Thermo Scientific, Waltham, MA, USA) for total RNA isolation, vortexed, and left on ice for a few minutes before being stored at −80 °C. The Illumina TruSeq Stranded mRNA protocol (Illumina, Inc., San Diego, CA, USA) was used to process the PVN RNA. The PIT, AMYG, ADC, and ADM sample tissues were cut and weighed to be between 130 and 160 mg, placed in RNase-free sterile cryovials, and stored at −80 °C until analysis. The frozen tissue samples were submitted to the Texas A&M Institute for Genome Sciences and Society (TIGSS) Experimental Genomics Core for RNA isolation and sequencing. Total RNA was isolated with the TRIzol Plus RNA Purification Kit (Thermo Scientific, Waltham, MA, USA). The Illumina TruSeq Stranded Total RNA kit was used to process the PIT, AMYG, ADC, and ADM RNA. The purified RNA was quantified with a Qubit RNA Fluorometric Assay Kit (Thermo Scientific), and the RNA quality was assessed with the Agilent 4200 Tape Station System (Software 5.1; Agilent Technologies, Santa Clara, CA, USA). The Agilent RNA ScreenTape^®^ assay for eukarotic RNA with a nucleotide reference ladder (25, 200, 500, 1000, 2000, 4000, and 6000 nt) was used to obtain the RNA integrity number (RINe).

### 2.3. RNA Sequencing and Annotation

TIGSS processed and analyzed the PVN, PIT, and AMYG total RNA samples using the HS protocol of the Illumina TruSeq Stranded mRNA library preparation kit with a Dual Indexed RNA Adapter Plate (Illumina, Inc., San Diego, CA, USA). For the ADC and ADM total RNA samples, TIGSS utilized globin and ribosomal RNA depletion polyA selection for mRNA isolation with the TruSeq Stranded Total RNA Library Prep workflow with Ribo-Zero Globin (Illumina, Inc.). The library fragment sizes were determined by the expected size of the Illumina protocol. The final libraries were validated with an Agilent 4200 TapeStation which determined the average base pair size of the sample. Cluster generation and sequencing was performed on the NovaSeq 6000 Sequencing System in paired-end, 150 bp cycles (Illumina, Inc.).

Raw gene counts were generated from the resulting RNA-Seq FastQ files by trimming the read adapters with Trimmomatic 0.38 [28]. The trimmed reads were then mapped to a *B. taurus* reference genome (Umd3.1), and the resulting mapped reads were counted using featureCounts 1.22.2 [29]. After generation of raw gene counts, the counts for each individual animal were combined into a single comma-separated file for each tissue with the Ensembl gene ID in the first column. These files were then used for further analysis with the edgeR Package (v. 3.13) from Bioconductor in R [30]. The raw gene count tables were read into rStudio (v. 4.2.2), and the low-expression genes were filtered out using the criteria of 3 minimum reads per gene in at least 5 samples. The normalization factors were then calculated using the trimmed mean of M values (TMM) method to produce normalized gene counts within each sample tissue.

### 2.4. Statistical Analyses

The normalized gene counts of genes important for circadian rhythms were extracted, adjusted using the Poisson distribution, and analyzed within each tissue of the five-year-old Brahman cows using the GLIMMIX and CORR procedures of SAS (SAS Institute, Cary, NC, USA). Analysis using the same SAS procedures was also performed to determine the statistical difference in expression for each gene between the tissues of the stress axis. Data are reported herein as least squares means contrasted using the Tukey modification as post hoc analysis when a significant F value (*p* < 0.05) was detected.

## 3. Results

**Figure 1:**
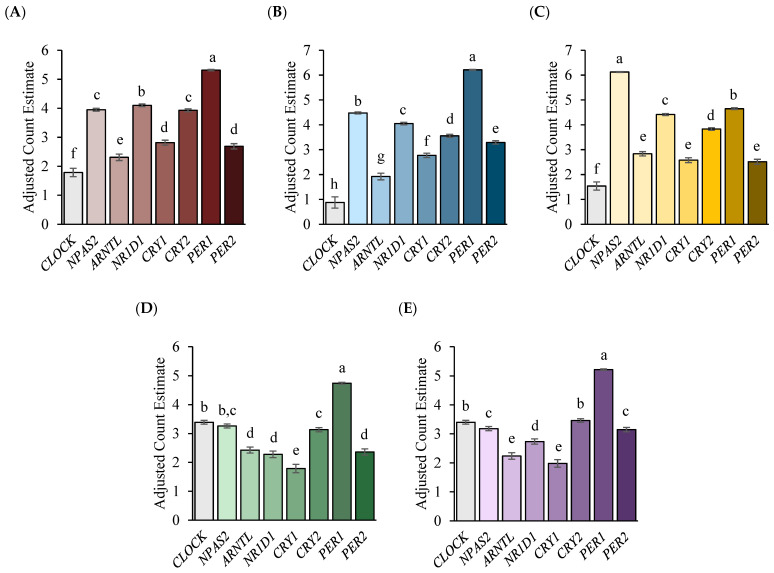
Comparison of the mean normalized circadian clock gene counts within the (**A**) PVN, (**B**) PIT, (**C**) AMYG, (**D**) ADC, and (**E**) ADM tissues of the stress axis. Counts were normalized using the Poisson distribution as described in the methods section. ^a, b, c, d, e, f, g, h^ Counts bearing differing superscripts differ from each other (*p* < 0.01).

Overall, the relative expression profiles of circadian clock genes differed (*p* < 0.01) both within and among the sample tissues of the stress axis, with no gene having zero counts. The expression of *PER1* was greater (*p* < 0.01) than the expression of the other circadian clock genes within the PVN, PIT, ADC, and ADM. After *PER1*, the main circadian clock genes that exhibited greater expression included *CLOCK*, *CRY2*, *NPAS2*, and *NR1D1*, depending on the sample tissue. Within the PVN, *NR1D1*, followed by *NPAS2* and then *CRY2*, was increased (*p* < 0.01; Figure 1A), while *NPAS2*, followed by *NR1D1*, was increased (*p* < 0.01) in the PIT (Figure 1B). For the adrenal tissues, the ADC exhibited greater expression (*p* < 0.01) of *CLOCK*, followed by *NPAS2* and then *CRY2* (Figure 1D), while within the ADM, the greatest expression (*p* < 0.01) was seen with the *CLOCK* and *CRY2* genes (Figure 1E). Similar to the other sample tissues, *NPAS2* expression within the AMYG was greatest relative to the expression of the other circadian clock genes (Figure 1C). Following *NPAS2*, *PER1* and then *NR1D1* exhibited greater expression (*p* < 0.01) within the AMYG.

**Figure 2:**
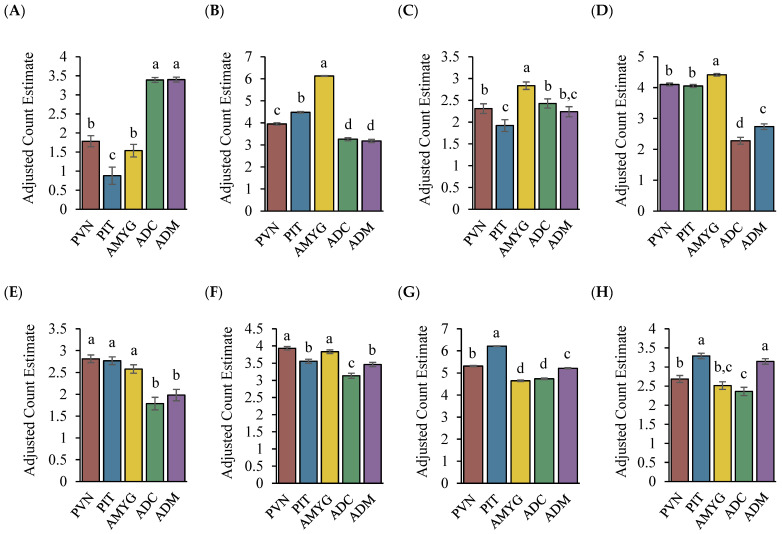
Comparison of the mean normalized gene counts for the (**A**) *CLOCK*, (**B**) *NPAS2*, (**C**) *ARNTL*, (**D**) *NR1D1*, (**E**) *CRY1*, (**F**) *CRY2*, (**G**) *PER1*, and (**H**) *PER2* circadian clock genes among each sample tissue of the stress axis. Counts were normalized using the Poisson distribution as described in the methods section. ^a, b, c, d^ Counts bearing differing superscripts differ from each other (*p* < 0.05).

As depicted in Figure 2A, *CLOCK* was the only circadian clock gene that had greater (*p* < 0.01) expression within both adrenal sample tissues, while *PER2* had greater (*p* < 0.01) expression in the ADM as well as the PIT (Figure 2H). The PIT also had greater expression (*p* < 0.01) of *PER1* compared with the other sample tissues (Figure 2G). Along with the PVN and AMYG, the PIT had greater expression (*p* < 0.01) of *CRY1* (Figure 2E). For the expression of *CRY2*, the PVN and AMYG exhibited greater (*p* < 0.01) expression relative to the other sample tissues (Figure 2F). Lastly, as depicted in Figure 2B–D, the AMYG exhibited greater (*p* < 0.01) expression of *NPAS2*, *ARNTL*, and *NR1D1*, respectively.

The circadian clock gene Pearson correlations and associated *p* values for each tissue of the stress axis are presented in Table 2 and Table 3. The PVN and PIT shared opposite correlation patterns (*p* < 0.05) between *NPAS2* and *NR1D1* (r = −0.77 and r = 0.89, respectively). With the exception of *NR1D1* (r = 0.89) within the PIT, *NPAS2* was negatively correlated (*p* < 0.05) with other circadian clock genes within the stress axis tissues. In the AMYG, there were positive correlations (*p* < 0.05) between *CLOCK* and *CRY2* (r = 0.92), *CLOCK* and *ARNTL* (r = 0.81), as well as *CRY2* and *ARNTL* (r = 0.85), while *CRY2* and *ARNTL* were negatively correlated with *NPAS2* (r = −0.82 and −0.74, respectively). There were no commonalities for the correlations within the adrenal tissues or between the ADC and other stress axis tissues. Significant correlations within the ADM followed similar correlation patterns to those for the PIT, with *PER1* being positively correlated (*p* < 0.05) with *CRY2* (r = 0.74 and r = 0.83, respectively) and *PER2* (r = 0.81 and r = 0.82, respectively). Additionally, *CRY2* was negatively correlated (*p* < 0.05) with *NPAS2* within both the ADM and PIT (r = −0.76 and r = −0.72, respectively). Although correlation analysis provides some evidence of correspondence (i.e., a positive or inverse relationship), a correlation coefficient does not prove causality.

## 4. Discussion

This study provides an initial demonstration of the expression of circadian clock genes within the bovine neuroendocrine tissues responsible for regulating stress responses, in addition to demonstrating differing expression of the circadian clock genes both within and among these sample tissues. Although most circadian rhythm research has focused on the SCN due to its role as the central pacemaker, the cells of peripheral tissues contain their own intrinsic circadian clock. This peripheral clock is reliant on the oscillating expression of core clock genes whose timing is mainly coordinated by the SCN, as well as glucocorticoids produced in the adrenal cortex [31,32]. However, longitudinal studies within the same animal of stress tissue gene expression in vivo are limited due to tissue inaccessibility, making the analysis of circadian rhythms in these tissues throughout a 24-h cycle difficult. Thus, the sample tissues of each animal for this study were harvested at a single timepoint. Despite this potential limitation, the analysis presented in this study presents a novel snapshot of bovine circadian clock gene expression in tissues other than the immune, liver, and mammary cells of cattle [19,22,33].

When comparing the top four expressed circadian clock genes within the cranial (PVN, PIT, and AMYG) and adrenal (ADC and ADM) stress tissues, three of the four clock genes (*PER1*, *NPAS2*, and *CRY2*) were common across all sample tissues. In each sample tissue, the expression of *PER1* was greater than the expression of the other circadian clock genes, with the exception of the AMYG and its expression of *NPAS2*. The PER1 protein translated from its respective mRNA is responsible for dimerizing with CRY proteins to primarily repress its own transcription through a transcription-translation negative feedback mechanism. However, PER1 protein can also regulate the expression of tissue-specific, clock-controlled genes outside of this regulatory feedback loop [34]. According to Chun et al. [32], increased amounts of *PER1* mRNA is potentially related to the ability of peripheral molecular clocks to adapt to environmental stimuli such as stress due to the presence of glucocorticoid response elements in the promoter sequence of the *PER1* gene [35]. Studies in rodent fibroblasts, livers, and forebrains (PVN, amygdala, and hippocampus) support this conjecture, as glucocorticoids rapidly induced *PER1* expression in these tissues [36,37]. Nebzydoski et al. [22] also showed an increase in *PER1* mRNA levels in the neutrophils and lymphocytes of cattle exposed to treatment with the synthetic glucocorticoid dexamethasone. The results of these studies, as well as the presence of the glucocorticoid response element on *PER1*, demonstrate the close relationship between glucocorticoid signaling and circadian *PER1* expression, which potentially explains the significantly greater expression of *PER1* in the stress-related tissues analyzed in our study.

For the PIT specifically, *PER1* expression was at least two times greater than its expression in the other sample tissues, which could potentially be attributed to its fundamental role in endocrine physiology. Observations in rodent [38], rhesus macaque [39], and human [40] cultured pituitaries showed a 24-h variation in *PER1* transcripts, demonstrating the presence of an intrinsic molecular clock. Bur et al. [38] showed that murine pituitary samples had a six-fold increase in *PER1* expression compared with murine liver samples. Though a direct comparison of core clock genes between the tissues of the stress axis has yet to be performed, it is clear that the expression of these genes, particularly *PER1*, is essential for controlling daily hormonal fluctuations, including those responsible for mediating stress response [12].

It is of interest to mention that the PIT as well as the ADM also exhibited greater expression of *PER2* compared with the PVN, AMYG, and ADC. While *PER1* expression is influenced by glucocorticoid response elements in its promoter sequence, *PER2* is influenced by glucocorticoids through an intron binding site of the *PER2* gene [35]. This mediation by glucocorticoids of *PER1* and *PER2* mRNA levels can modulate the rhythmic influence of peripheral clocks during stressful events to enhance stress response.

Though *PER1* in the AMYG did not exhibit the greatest level of expression relative to the other circadian clock genes, its expression was still at a similar level to *PER1* expression in the other sample tissues, except for the PIT. Instead, the expression of *NPAS2* in the AMYG was at least four times greater than the expressions of the other circadian clock genes, as well as being at least five times greater than *NPAS2* expression in the other sample tissues. As a paralogue of *CLOCK*, the NPAS2 protein encoded by the *NPAS2* gene can dimerize with ARNTL to activate the transcription of the negative regulatory *PER* and *CRY* genes, as well as the transcription of clock-controlled genes [9,41]. Similar to the other circadian clock genes, *NPAS2* expression can be found in both the SCN and peripheral tissues. If there is deficient CLOCK, then NPAS2 can play a compensatory role to maintain a circadian rhythm, particularly in neural tissues such as the SCN, PVN, and AMYG as well as the PIT [10].

In addition to its role in circadian rhythms, *NPAS2* is also responsible for regulating fear and anxiety behavior along with memory learning [41,42], which are some of the primary functions of the AMYG [43,44,45]. Thus, the extensive expression of *NPAS2* in the AMYG relative to the other circadian clock genes and sample tissues could be explained by the mutual regulatory role of NPAS2 and the AMYG in memory and learning. The expression of *ARNTL* in the AMYG also was greater relative to the other tissues sampled. This gene is an essential circadian clock gene that produces a core component of the circadian clock, ARNTL protein, which along with NPAS2 protein regulates physiological processes, including emotional processing and memory formation [46,47].

One of the major differences between the cranial and adrenal tissues is the expression of *CLOCK* and *NR1D1*, where the ADC and ADM had similar levels of *CLOCK* expression that were greater (*p* < 0.01) than those in the cranial tissues, while the PVN, PIT, and AMYG had greater (*p* < 0.01) expression levels of *NR1D1* than those in both adrenal tissues. The nuclear receptor REV-ERBα encoded by *NR1D1* is another core component of the transcription-translation negative feedback mechanism. Unlike PER and CRY, REV-ERBα plays a dual role in regulating circadian rhythms by suppressing *ARNTL* and *CLOCK* expression through its binding sites within the promoter and intron regions of these genes, respectively [8,34]. This suppression contributes to the rhythmic control of the negative regulatory loop generated by the circadian clock genes. Thus, a potential theory for this discrepancy in *NR1D1* and *CLOCK* expression between the cranial and adrenal sample tissues could be that they are in different phases of the circadian rhythm, possibly because of a delay in circadian signals from the central pacemaker reaching the adrenal tissues relative to the cranial tissues due to their proximity to the SCN [48].

Within the adrenal gland, particularly the cortex, the expression of *CLOCK* mediates the rhythmic production of glucocorticoids by controlling the expression of steroidogenic acute regulatory protein (StAR), a mitochondrial protein that facilitates the movement of cholesterol into the mitochondria for steroidogenesis [49]. This additional role of *CLOCK* within the adrenal tissues could explain the increased expression of *CLOCK* in both the ADC and ADM relative to the other sample tissues. Like *CLOCK*, *NR1D1* also has important functions in addition to its negative regulatory role in circadian rhythms, where *REV-ERBα* regulates metabolism, memory, and behavior [50,51], plays a role in muscle and cartilage formation [52,53], and contributes to inflammatory response [54,55]. The expression of *NR1D1* in the brain is also essential during neurodevelopment, as deficiencies in this gene have been associated with neurological disorders such as autism spectrum disorder. Knockdown of this gene in mice led to the abnormal growth of neurons in the cerebral cortex [56], potentially explaining the elevated expression of *NR1D1* in the cranial sample tissues relative to the adrenal tissues analyzed in this study.

Another potential explanation for the reduced expression of *NR1D1* in the adrenal tissues could be that the production of glucocorticoids in the adrenal gland and the presence of their corresponding receptor suppresses *NR1D1* expression. Torra et al. [57] found that the hepatic expression of *NR1D1* in mice was reduced by 70% after glucocorticoid treatment. This reduction could potentially be explained by the repressive mechanism proposed by Murayama et al. [58], where the glucocorticoid receptor (GR) forms a complex with the CLOCK-ARNTL heterodimer that binds to the promoter region of *NR1D1* and, through this GR-CLOCK-ARNTL complex, indirectly suppresses *NR1D1* transcription. However, further investigation is needed to determine the mechanism responsible for the discrepancy of *CLOCK* and *NR1D1* expression in the cranial and adrenal stress tissues.

Although not extensively different among tissues relative to the other circadian clock genes, *CRY1* was also reduced in the adrenal tissues (i.e., the adrenal cortex and adrenal medulla) relative to the cranial sample tissues (i.e., the paraventricular nucleus, amygdala, and anterior pituitary). The CRY1 protein encoded by *CRY1* has the same negative regulatory role as *PER* in terms of mediating circadian rhythms, although unlike *PER*, *CRY1* is also responsible for promoting DNA repair and cell survival upon genomic insult through temporal transcriptional regulation. This function of *CRY1* has mainly been focused upon in cancer research using both rodent and human sample cells [59,60]. Similar to the expression difference of *CLOCK* and *NR1D1*, it is not clear whether the difference in *CRY1* expression between the cranial and adrenal sample tissues is due to SCN proximity or an additional gene function outside of mediating circadian rhythms. Further investigation into the expression of circadian clock genes in the stress tissues over a 24h period would help provide an explanation for the clock gene expression differences observed in these data, but there are serious physical challenges that preclude monitoring the cells of these tissues *in situ* in real time. Experimental approaches such as cell cultures or simultaneous sampling of tissue-specific endocrine products and serial sampling of animals may become feasible and informative.

## 5. Conclusions

The concatenation of findings from basic research on various animals and clinical studies of people supports the concept of a physiological linkage of circadian rhythms and the adrenal axis [61,62]. The genetic mechanism controlling this bidirectional communication hinges on the genes coding for the glucocorticoid receptor and clock genes (central and peripheral). The potential role of micro RNAs in regulation of the expression of the glucocorticoid receptor and clock genes (central and peripheral) merits investigation with in vitro models [63]. The study of circadian rhythms is necessary, as these rhythms play a vital role in preparing an organism’s body for expected changes in the environment within a 24 h period. It is important for controlling various physiological processes, including the regulation of sleep, metabolism, stress response, blood pressure, body temperature, and the immune system [64,65]. The ontogeny of the coordinated entrainment of circadian rhythms and the HPA axis is an emergent investigational area [66,67]. This topic is of importance in light of the important health role of circadian rhythms from infancy throughout one’s lifespan [61]. Several limitations impede studies to assess circadian rhythms and clock gene expression in livestock species. Nevertheless, there has recently been an increase in research focusing on circadian rhythm regulation of livestock physiology. Porcine, ovine, and bovine research animals can be the platform for investigating the effects of fetal programming or early life adversity effects on the maternal-fetal circadian gene system. The aging equine model provides a vehicle through which to acquire new insight regarding longevity, health, the HPA axis, and circadian rhythms. Our study gives a first look at circadian clock gene expression within the stress axis tissues of cattle, providing the first step in understanding the intimate connection between circadian rhythms and stress response needed for improving livestock husbandry and welfare. Future studies focusing on the temporal relationships of these genes with the primary endocrine products of the stress tissues should be conducted to define the roles of peripheral clock genes in the regulation of cattle metabolism, stress response, and immune health.

## Figures and Tables

**Table 1:** Genes involved in the regulation of circadian rhythms (https://useast.ensembl.org, accessed on 1 November 2023).

Common Name	Gene Symbol	Ensembl Number
Circadian locomotor output cycles protein kaput	*CLOCK*	ENSBTAG00000044044
Neuronal PAS domain protein 2	*NPAS2*	ENSBTAG00000019697
Aryl hydrocarbon receptor nuclear translocator like	*ARNTL*	ENSBTAG00000013029
Nuclear receptor subfamily 1 group D member 1	*NR1D1*	ENSBTAG00000012178
Cryptochrome circadian regulator 1	*CRY1*	ENSBTAG00000010149
Cryptochrome circadian regulator 2	*CRY2*	ENSBTAG00000021223
Period circadian regulator 1	*PER1*	ENSBTAG00000003889
Period circadian regulator 2	*PER2*	ENSBTAG00000001879

**Table 2:** Pearson correlations (above the diagonal line) and associated *p* values (below the line) between circadian clock genes within each cranial tissue of the stress axis. Significant correlations and their associated *p* values are in bold font.

**PVN**	*CLOCK*	*NPAS2*	*ARNTL*	*NR1D1*	*CRY1*	*CRY2*	*PER1*	*PER2*
*CLOCK*	-	**−0.737**	0.421	0.356	−0.256	0.125	−0.646	0.006
*NPAS2*	**0.037**	-	−0.643	**−0.769**	0.337	−0.558	0.174	−0.434
*ARNTL*	0.299	0.085	-	0.447	**−0.778**	0.316	−0.442	−0.185
*NR1D1*	0.386	**0.026**	0.267	-	−0.173	**0.852**	0.215	0.548
*CRY1*	0.540	0.414	**0.023**	0.681	-	−0.091	0.298	0.349
*CRY2*	0.767	0.151	0.446	**0.007**	0.830	-	0.265	0.323
*PER1*	0.084	0.680	0.273	0.609	0.474	0.527	-	0.652
*PER2*	0.989	0.283	0.662	0.160	0.397	0.435	0.080	-
**PIT**	*CLOCK*	*NPAS2*	*ARNTL*	*NR1D1*	*CRY1*	*CRY2*	*PER1*	*PER2*
*CLOCK*	-	−0.595	0.071	−0.414	**0.710**	0.285	−0.208	−0.223
*NPAS2*	0.120	-	0.297	**0.892**	−0.663	**−0.718**	−0.298	−0.232
*ARNTL*	0.868	0.475	-	−0.025	0.449	−0.680	**−0.723**	−0.614
*NR1D1*	0.308	**0.003**	0.953	-	**−0.789**	−0.471	−0.065	−0.066
*CRY1*	**0.048**	0.073	0.263	**0.020**	-	0.154	−0.349	−0.230
*CRY2*	0.494	**0.045**	0.063	0.239	0.716	-	**0.830**	0.701
*PER1*	0.620	0.474	**0.043**	0.878	0.397	**0.011**	-	**0.818**
*PER2*	0.596	0.581	0.106	0.877	0.584	0.053	**0.013**	-
**AMYG**	*CLOCK*	*NPAS2*	*ARNTL*	*NR1D1*	*CRY1*	*CRY2*	*PER1*	*PER2*
*CLOCK*	-	−0.670	**0.813**	−0.331	0.537	**0.917**	−0.120	0.171
*NPAS2*	0.054	-	**−0.741**	−0.214	−0.251	**−0.821**	−0.173	−0.364
*ARNTL*	**0.014**	**0.035**	-	−0.162	0.458	**0.854**	0.058	0.500
*NR1D1*	0.424	0.611	0.701	-	−0.215	−0.173	0.246	0.570
*CRY1*	0.170	0.548	0.253	0.610	-	0.277	0.055	0.013
*CRY2*	**0.001**	**0.012**	**0.007**	0.681	0.506	-	−0.079	0.351
*PER1*	0.778	0.682	0.891	0.558	0.896	0.852	-	−0.157
*PER2*	0.686	0.375	0.207	0.140	0.976	0.394	0.710	-

**Table 3:** Pearson correlations (above the diagonal line) and associated *p* values (below the line) between circadian clock genes within each adrenal tissue of the stress axis. Significant correlations and their associated *p* values are in bold font.

**ADC**	*CLOCK*	*NPAS2*	*ARNTL*	*NR1D1*	*CRY1*	*CRY2*	*PER1*	*PER2*
*CLOCK*	-	−0.577	0.625	−0.494	−0.052	0.362	0.100	0.197
*NPAS2*	0.135	-	−0.540	0.636	0.145	−0.366	0.016	0.485
*ARNTL*	0.098	0.167	-	**−0.738**	0.421	0.377	−0.363	−0.291
*NR1D1*	0.213	0.090	**0.037**	-	−0.198	−0.148	0.149	−0.329
*CRY1*	0.902	0.732	0.299	0.638	-	0.021	0.217	**0.734**
*CRY2*	0.379	0.372	0.357	0.726	0.961	-	0.691	0.561
*PER1*	0.815	0.377	0.969	0.725	0.606	0.058	-	0.649
*PER2*	0.640	0.485	0.223	0.426	**0.038**	0.148	0.081	-
**ADM**	*CLOCK*	*NPAS2*	*ARNTL*	*NR1D1*	*CRY1*	*CRY2*	*PER1*	*PER2*
*CLOCK*	-	−0.049	0.469	−0.237	−0.206	−0.243	−0.301	−0.450
*NPAS2*	0.909	-	−0.405	−0.703	−0.365	**−0.762**	−0.510	−0.332
*ARNTL*	0.241	0.320	-	−0.003	0.283	0.422	0.164	0.277
*NR1D1*	0.571	0.052	0.995	-	0.066	0.395	0.560	0.402
*CRY1*	0.625	0.374	0.497	0.876	-	0.438	0.095	0.341
*CRY2*	0.563	**0.028**	0.298	0.333	0.278	-	**0.742**	0.564
*PER1*	0.468	0.197	0.697	0.149	0.822	**0.035**	-	**0.807**
*PER2*	0.264	0.422	0.506	0.324	0.409	0.145	**0.015**	-

## Data Availability

Data will be made available upon reasonable request.
